# Impacts of Agricultural Pesticide Contamination: An Integrated Risk Assessment of Rural Communities of Eswatini

**DOI:** 10.3390/toxics11090770

**Published:** 2023-09-10

**Authors:** Sithembiso Sifiso Msibi, Lihchyun Joseph Su, Chung-Yu Chen, Cheng-Ping Chang, Chiou-Jong Chen, Kuen-Yuh Wu, Su-Yin Chiang

**Affiliations:** 1 O’Donnell School of Public Health, University of Texas Southwestern Medical Center, 5323 Harry Hines Boulevard, Dallas, TX 75390, USA; sithembiso.msibi@utsouthwestern.edu (S.S.M.); lihchyun.su@utsouthwestern.edu (L.J.S.); 2 Department of Occupational Safety and Health, College of Health Sciences, Chang Jung Christian University, No. 1, Changda Rd., Guiren District, Tainan 71101, Taiwan; cyuchen@mail.cjcu.edu.tw (C.-Y.C.); jimmycpc@mail.cjcu.edu.tw (C.-P.C.); akichn@mail.cjcu.edu.tw (C.-J.C.); 3 Institute of Food Safety and Health, College of Public Health, National Taiwan University, No. 17, Xuzhou Rd., Taipei 10055, Taiwan; 4 Department of Public Health, College of Public Health, National Taiwan University, No. 17, Xuzhou Rd., Taipei 10055, Taiwan; 5 Institute of Environmental and Occupational Health Sciences, College of Public Health, National Taiwan University, No. 17, Xuzhou Rd., Taipei 10055, Taiwan; 6 School of Chinese Medicine, College of Chinese Medicine, China Medical University, No. 91, Hsueh-Shih Rd., Taichung 40402, Taiwan

**Keywords:** pesticides, agriculture, liquid chromatography with tandem mass spectrometry, environmental media, multimedia model, health risk, ecological risk, Eswatini

## Abstract

Marked reductions in mean annual rainfall associated with climate change in Eswatini in Southern Africa have encouraged the recycling of irrigation water and the increased use of pesticides in agricultural production, raising concerns about potential ecological and health risks due to long-term exposure to pesticide residues in soil and irrigation water. This probabilistic integrated risk assessment used liquid chromatography with tandem mass spectrometry to analyze the concentrations of four commonly used agricultural pesticides (ametryn, atrazine, pendimethalin, and 2,4-dichlorophenoxyacetic acid (2,4-D)) in irrigation water and topsoil samples from farmlands in Eswatini to assess potential ecological and health risks due to exposure. The concentrations of these pesticides ranged from undetectable to 0.104 µg/L in irrigation water and from undetectable to 2.70 µg/g in soil. The probabilistic multi-pathway and multi-route risk assessments conducted revealed hazard indices exceeding 1.0 for all age groups for ametryn and atrazine, suggesting that the daily consumption of recycled irrigation water and produce from the fields in this area may pose considerable health risks. The indices pertaining to ecological risks had values less than 0.1. Adaptation measures are recommended to efficiently manage pesticide use in agriculture, and further research will ensure that agriculture can adapt to climate change and that the general public and ecosystem are protected.

## 1. Introduction

The intensive use of pesticides in agricultural production has led to their ubiquitous presence in the environment. Approximately 3 million tons of pesticides valued at USD 40 billion are sprayed annually worldwide [1]; Africa accounts for a mere 2–4% of the global market [2]. Between 1990 and 2017, the amount of agricultural pesticides used throughout Africa increased by 26% [3]. This trend is projected to continue, especially in sub-Saharan Africa, as most African countries still rely heavily on agricultural production for economic growth [4], so the importance of agricultural pesticides for countries in Africa cannot be overemphasized. However, serious concerns surround the potential ecological and health risks associated with pesticide use [5,6].

Due to the lack of adequate rainfall for agricultural production in Africa, this continent is experiencing a serious water shortage crisis associated with the effects of climate change. Additional problems include competition for scarce water resources amongst communities, pollution, and inefficient management of water resources [7]. Many African countries face huge challenges in providing safe drinking water for communities [8]. Eswatini in Southern Africa has experienced a decline of 20% in mean annual rainfall between 1986 and 2017 [9]. Notably, during the 2015/2016 growing season, the amount of rainfall fell by as much as 50% compared to the previous season [10]. The existing water reservoirs and water transfer schemes cannot fully satisfy the demands for irrigation and domestic use, especially in rural areas. Importantly, sugarcane is the most important cash crop in Eswatini, accounting for 10% of the country’s annual gross domestic product (GDP) [11]. Sugarcane production and the use of pesticides increased by 14% between 2010 and 2019 [3], without any commensurate increase in water supply, which is needed for sugarcane cultivation.

The increased use of agricultural pesticides heightens health concerns for residents in rural communities. Spray drift during pesticide application in fields enables a fraction of the applied pesticides to be emitted into the atmosphere [12], while pesticides in the soil may contaminate water sources via run-off from agricultural lands [13,14]. Thus, the environment around farmlands is susceptible to pesticide contamination, and the resulting human exposure, particularly through the production and consumption of agricultural produce, is of enormous concern. The relative contribution of each environmental source can be challenging to discern, as human exposure can occur through inhalation, ingestion, and dermal pathways, any of which can result in acute and chronic adverse health effects [15,16].

Even at very low levels, pesticide exposure adversely affects health for all age groups, but it particularly affects young children, with reports of autism spectrum disorders and neurodevelopmental delays associated with their exposure to pesticides [17,18]. Chronic pesticide exposure is associated with deleterious health effects such as hormonal imbalances, asthma, various allergies, and cancer [13,19,20,21,22,23]. It is estimated that 70% of the population of Eswatini resides in rural areas [24], with commercial and subsistence farming being common activities in their local communities, so most of the country’s inhabitants are exposed to pesticides through environmental media. Thus, it is important to assess the exposure of the rural population to multiple environmental media and the resulting health risks. This investigation set out to conduct a probabilistic cumulative risk assessment of residents in an Eswatini agricultural community. These people are exposed to four commonly used pesticides: ametryn, atrazine, pendimethalin, and 2,4-dichlorophenoxyacetic acid (2,4-D). Research has shown that exposure to these pesticides has deleterious effects on environmental and human health. Exposure to atrazine is associated with an increased risk of pre-term births [25] and irregular menstrual cycles [26] among women. Studies have found an increased risk of non-Hodgkin lymphoma among people exposed to 2,4-D [27], while others showed no association [28]. In addition, some studies have found evidence suggesting that there is an association between 2,4-D exposure and an increased risk of prostate and gastric cancer [29,30]. Ametryn exhibits low levels of acute and chronic toxicity in humans [31], and a recent computational analysis confirmed the probability of pendimethalin acting as an antiandrogen due to its endocrine disrupting potential [32].

As there are no systematic exposure and risk assessments for agricultural pesticides in soil and water in Africa, this study aimed to assess, for the first time, the potential ecological and health risks arising from pesticide exposure for residents of agricultural communities in Eswatini. Using liquid chromatography and tandem mass spectrometry, we analyzed the concentrations of four pesticides in irrigation water, topsoil, and air samples from the farmlands of Eswatini. The quantification of those chemicals in environmental media (air, soil, and water) was integrated using the CalTOX multimedia exposure model and compared with a toxicological reference dose of the target compound to estimate hazard indices (HIs). Using Eswatini as an example, this case study sought to highlight concerns for human health and ecological risks contributed by agricultural pesticide residues in recycled irrigation water in order to address the potential impact of climate change on many African countries.

## 2. Materials and Methods

### 2.1. Study Area

**Figure 1:**
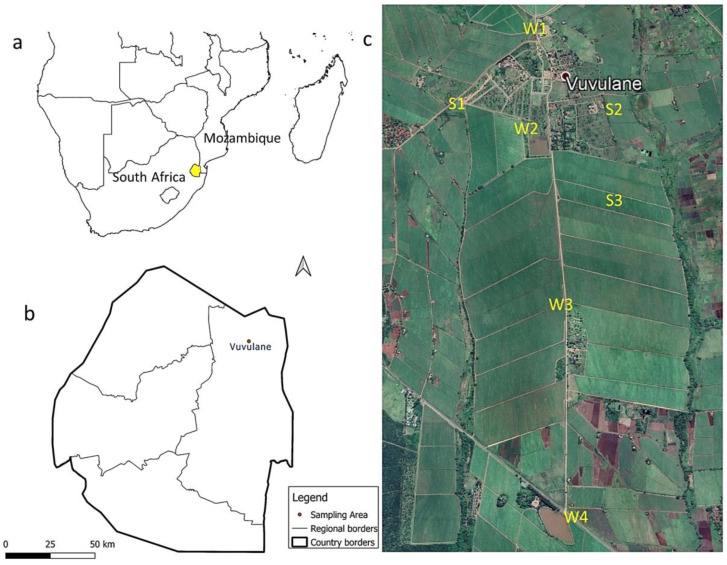
Overview maps of the study area. (**a**) A geographical map of Southern Africa showing the location of Eswatini; (**b**) a map of Eswatini showing the location of the study area; (**c**) the distribution map of the soil (S) and water (W) sampling points in the study area.

The study area, Vuvulane, is an agricultural community located on a commercial sugarcane farm in the northeastern part of Eswatini (Figure 1). It is estimated that 70% of Eswatini’s area is agricultural land [33], with sugarcane production being an important part of its economy. The commercial farm in Vuvulane relies on local canal irrigation systems to grow sugarcane throughout the year and supply the country’s largest sugar mill. The studied community is situated at 26.07° S and 31.87° E, with an elevation of 273 m above sea level, in the arid Lowveld region of Eswatini. This region receives the lowest yearly amount of rainfall (annual mean 400 mm) in the country, with temperatures of 37 °C in summer and 7 °C in winter [33]. Water for irrigation and domestic purposes in this community is sourced from the Sand River Dam. Water is transferred through canals and pipes to the fields and the community. The commercial farm in this study has an estimated area of around 14 hectares and practices extensive pesticide application throughout the growing season to maintain high yields and crop quality. Although the applicators are aware of the recommended application rates provided in the product information sheets, they may use higher amounts than the recommended doses, as our previous paper revealed high levels of inhalation exposure amongst applicators on the studied farm due to the amounts sometimes used [34].

### 2.2. Field Sampling

#### 2.2.1. Soil Sampling

A total of 10 topsoil samples (500 g each) were collected from sugarcane fields during the spraying season in December 2018. The selection of sampling sites within the field was carried out using stratified random sampling, as pesticides are not uniformly applied. Some areas in the field would therefore have higher concentrations of pesticides. The field was divided into three subunits. An auger was used to collect soil samples. In each subunit, eight subsamples were collected (at depths of 0–15 cm) in a zigzag pattern with 5–10 m intervals. The subsamples were combined into a homogenous mix in a jar, and foreign materials such as plant roots, stones, and leaves were removed. A composite sample (500 g) was collected through mixing and compartmentalizing the subsamples. The mixed sample was divided into 4 compartments, and only a single scoop of soil collected from each compartment was placed into a polythene bag. The procedures for mixing subsamples in a jar and pouring composite samples into bags were repeated until all samples were collected. Sterile gloves were used to prevent contamination during the sampling process. Samples were clearly labeled and transported to a laboratory in Kwaluseni, Eswatini, where they were refrigerated. They were then shipped in dry ice to Tainan, Taiwan, where they were kept at −20 °C until analysis.

#### 2.2.2. Water Sampling

Untreated irrigation water samples were collected at depths of 0–2 m from streams that the community primarily uses for irrigating their agriculture fields. Due to the water shortage in the community, this water is sometimes consumed by community members. We used systematic sampling, and a bucket and rope were used to collect water samples. Several equal-sized subsamples were collected and thoroughly mixed in a clean container. This composite sample was poured into a 1 L plastic bottle. Sample bottles were filled to the brim to eliminate any airspace, as the water–air interface may allow some chemicals to vaporize prior to analysis. Samples were systematically collected at four different points along the stream based on the distance from the nearest sugarcane field in the following order: <20 m, 21–50 m, 51–100 m, and 101–150 m. Twelve samples were collected, labelled clearly, and transported in ice chests to a laboratory in Kwaluseni, Eswatini. To prevent biodegradation of the analytes in the samples, the samples were acidified to pH3 using sulphuric acid before refrigeration. They were then shipped in dry ice to Tainan, Taiwan, where they were stored at −20 °C.

#### 2.2.3. Air Sampling

Air-sampling data used in this study, including personal pesticide exposure levels and indoor air pesticide concentrations, are cited in our previous publications [34,35].

### 2.3. Sample Preparation and Cleanup

Detailed information on the properties of the target compounds (ametryn, atrazine, pendimethalin, and 2,4-D), including their molecular weights, molecular formulas, log P values, and half-life times in days (DT_50_), are available in the Supplementary Material (Table S1). Cambridge Isotope Laboratories, Inc. (Tewksbury, MA, USA) supplied the internal standard atrazine (ethylamine-D5) that was incorporated at 100 µg/mL in nonane. A QuEChERS extraction kit (EN 15662 method [36]; P/N 5982-5650; 5982-5156) was supplied by Agilent Technologies (Santa Clara, CA, USA). Oasis HLB 500 mg/6 mL Vac Cartridges were purchased from Waters Corporation (Milford, MA, USA). We used the QuEChERS approach for extracting pesticide residues from the soil samples, adhering to the guidelines issued by the European Commission for Standardization (EN 15662:2018) [36]. A portion of the 500 g sample was air-dried at about 30 °C. A total of 5 g of the soil sample was deposited into a 50 mL centrifuge tube spiked with 10 µL internal standard at 25 mg/L. The mixture was shaken by hand for 1 min to produce a homogeneous mixture before adding 10 mL of acetonitrile, methanol, and deionized water (5:4:1 *v*/*v*) with 1% acetic acid. The mixture was supplemented with ready-to-use sachets of 4 g of anhydrous magnesium sulfate (MgSO_4_), 1 g of sodium acetate (NaOAc), and a ceramic homogenizer. It was then vortexed at 1000 revolutions per min (rpm) for 2 min to prevent the formation of crystalline agglomerates and centrifuged at 15 °C at a relative centrifugal force (RCF) of 4000 for 1 min using a Universal 320 R centrifuge (Hettich Group, Tuttligen, Germany). For the cleanup process, 6 mL of the supernatant was transferred into a 15 mL centrifuge tube containing 900 mg of MgSO_4_, 150 mg of PSA, and 150 mg of C18. The mixture was vortexed at 1000 rpm for 1 min and centrifuged at 5549 rpm for 2 min at 15 °C. The supernatant was transferred into a 15 mL tube and concentrated to dryness at 40 °C for 1 h 30 min using a Savant SPD1010 concentrator (Thermo Fisher Scientific, Waltham, MA, USA). The residue was redissolved in 1 mL of methanol, filtered through a polytetrafluoroethylene (PTFE) membrane filter (0.22 µm), and subsequently analyzed using liquid chromatography with tandem mass spectrometry (LC-MS/MS).

A 250 mL aliquot of the water sample was first filtered through 0.45 µm filter paper to remove particulates and debris and then processed through solid phase extraction (SPE). Oasis HLB (500 mg, 6 mL) sorbents were used to extract the target compounds. The sorbents were conditioned with 10 mL of methanol and 10 mL of pure water. The samples were spiked with the internal standard and then loaded into the sorbent cartridges. Due to the different polarities of the target compounds, elution was performed using four different organic solvents; 2 mL each of dichloromethane, acetone, methanol, and ethyl acetate were sequentially added to the sorbents to elute the analytes. The eluates were subsequently evaporated to dryness under nitrogen steam. The residue was redissolved in 1.0 mL of methanol, water, and acetic acid (80:20:0.1 *v*/*v*), and the extract was filtered using a 0.22 µm PTFE membrane filter and analyzed using LC-MS/MS.

### 2.4. LC-MS/MS Analysis, Pesticide Determination, and Quality Control

The same chemicals, solvents, and LC-MS/MS analysis described in our previous paper were used in this study [34]. For quality control and ensuring the accuracy of our analysis, we used an internal standard (atrazine-d5) purchased from Cambridge Isotope Inc. (Andover and Tewksbury, MA, USA). Eight-point calibration curves for all analytes were derived in the range from 1 to 200 µg/L. Mean recoveries for all target compounds were determined using blank samples that were spiked, extracted, and analyzed under the same conditions as the field samples (see Table S2). Pesticide concentrations in soil samples were calculated using the following equation:

(1) $$C_{soil}=\frac{C_{a}\times V}{M}$$

In this equation, *C_soil_* is the concentration of pesticide in soil (µg/g), *C_a_* is the analyte concentration in the extract measured using LC-MS/MS (µg/L), *V* is the volume used in extraction (L), and *M* is the mass of the soil sample used in extraction (g). Pesticide concentrations in water samples were determined using the following equation:

(2) $$C_{water}=\frac{C_{a}\times V_{1}}{V_{sample}}$$

where *C_water_* is the final concentration of pesticides in water (µg/L), *C_a_* is the concentration of the analyte measured using LC-MS/MS (µg/L), *V*_1_ is the volume of the sample after redissolution (L), and *V_sample_* is the initial volume of water used for sample preparation (L). For calculating averages, we assigned a proxy value of half of the limit of detection (LOD) in samples for which the level of each analyte was not detectable (ND).

### 2.5. Multi-Pathway and Multi-Route Risk Assessments

**Figure 2:**
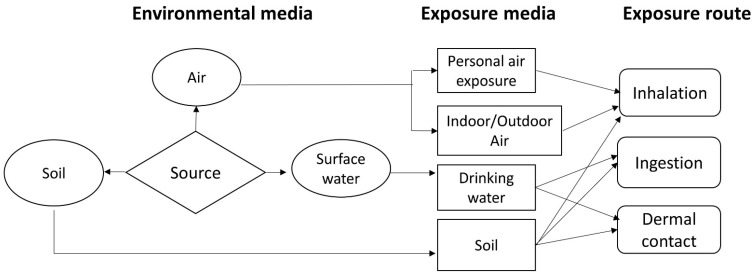
Exposure pathways in a multimedia model framework (Adapted from [41]).

This study assessed pesticide exposure via different environmental media and pathways using a probabilistic framework for multimedia and multi-pathway health risk assessments (Figure 2). Multimedia fate and exposure modeling can be applied to support site-specific assessments [37,38]. The CalTOX^TM^ 4.0 beta model is a multimedia total exposure model developed by the California Environmental Protection Agency to improve risk assessments and estimate the chemical fates of and human exposure to contaminants in air, soil, and water [39]. CalTOX has been applied for risk assessments of hazardous compounds, including organophosphorus pesticides [40]. In this study, the multimedia fate and exposure model was complemented by measurement data for assessing the risk of exposure in the study area.

### 2.6. Environmental Data and Exposure Factors

The health risk of chronic exposure to pesticide residues for residents in the agricultural community was assessed for four age groups (0–3 years, 4–12 years, 13–18 years, and 19–65 years). All target pesticides are non-carcinogenic (Table S3) based on classifications issued by the United States Environmental Protection Agency [42]. For probabilistic assessment, we used CalTOX software combined with Microsoft Excel™ 2016 and Oracle© Crystal Ball Release 11.1.2.4.00 software (Oracle Corporation, Austin, TX, USA). CalTOX is a multimedia, multiple pathway risk assessment model used to estimate the health risk faced by people living or working near a contaminated area [43]. It uses equations based on the conservation of mass and the chemical equilibrium principle, as it predicts the concentrations of chemicals and exposure doses via ingestion, inhalation, and dermal contact over a period of time [44]. We ran 1000 Monte Carlo (MC) simulations. All input parameters were assumed to follow log-normal distributions [45,46,47]. The amount of pesticide sprayed (mol/day) was based on the typical pesticide application rates (kg/ha) on the farm and the acreage (ha) of treated fields as reported in our previous paper [26]. The estimated amounts used of each pesticide were 123.2 mol/day for ametryn, 64.9 mol/day for atrazine, 62.2 mol/day for pendimethalin, and 41.8 mol/day for 2,4-D. The reference dose (RfD) used for each pesticide was 0.09 mg/kg/day for ametryn, 0.035 mg/kg/day for atrazine, 0.04 mg/kg/day for pendimethalin, and 0.01 mg/kg/day for 2,4-D (see Table S3). We used our environmental data measurements for the levels of pesticides in household indoor air, irrigation water, and agricultural soil samples. A model was applied to simulate the distribution of our target pesticides across the studied environmental media by assuming that there were continuous emissions. Table S4 lists the concentrations of the four target pesticides in different environmental media collected in Eswatini. We manually entered ametryn and pendimethalin into the CalTOX software, using their chemical properties, as they were not available on the list of chemicals in the model. For unknown environmental parameters, we adopted the built-in parameters from the State of Arizona in the USA. This was performed due to the similarity of the climatological conditions between this state and our study area, as both areas are in temperate regions. However, for known parameters such as the annual averages of ambient temperature, wind speed, precipitation, and land area, we used local values from our study area as model inputs (see Table S5). For exposure factors, we used values from the 2011 edition of the USEPA Exposure Factors Handbook [48]. Body weights for local residents in Eswatini were adopted from the Eswatini Government’s Disease Risk Factor Surveillance Report [49]. The exposure duration (ED) was assumed to be over a 60-year lifetime based on Eswatini’s life-expectancy-at-birth data [50]. The average time (AT) in days was estimated as ED (years) multiplied by 365 days/year. The exposure factors are summarized in Table S6. In order to rank the importance of influential parameters in the CalTOX model, we conducted a sensitivity analysis using Crystal Ball software.

### 2.7. Ecological Risk Assessment

The risk quotient (*RQ_i_*) was calculated using the measured environmental concentration in water (*MEC_i_*) and the predicted no-effect concentration (*PNEC_i_*) [51], where *i* = 1–4, representing ametryn, atrazine, pendimethalin, and 2,4-D. The *PNEC_i_* was derived using the conventional method, for which the assessment factor (*AF*) was used. Toxicity data were obtained from a toxicity database (www.epa.gov/ecotox, accessed on 15 January 2023); acute and chronic toxicity data were selected from freshwater single-species studies, focusing on fish and aquatic invertebrate species. The main water source (the Sand River Dam) for the studied community is an important artificial reservoir for threatened fish species. It has been recognized as a wetland of international importance [52] and is home to common fish species, including species of tilapia (including *Oreochromis mossambicus* and *Tilapia rendalli*) and catfish (*Clarias gariepinus*) [53,54]. Local residents occasionally fish in the reservoir for subsistence or commercial purposes. However, in recent times, this has been highly discouraged due to concerns over biodiversity conservation. The pattern of occurrence of aquatic invertebrate fauna is strongly seasonal, and invertebrates such as damselflies, earthworms, and insect larvae are found in the dam. When selecting the toxicity value, the geometric mean for all toxicity values was calculated. *RQ_i_* was calculated using the following equation [55]:

(3) $${RQ}_{i}=\frac{{MEC}_{i}}{{PNEC}_{i}}$$

*PNEC* was derived using the following equation [56]:

(4) $$\log\;(PNEC)=\log\;(\min\left(NOEC\right)-\log\;(AF)$$

where *AF* is the assessment factor and min (no-observed-effect concentration, *NOEC*) is the minimum *NOEC* obtained from the toxicity data set. Log (*AF*) = 1, since we assume that *AF* = 10 as there is at least one set of data for each taxonomic group. The risk values were classified as insignificant if *RQ_i_* < 0.1; as low risk if 0.1 ≤ *RQ_i_* < 1; as moderate risk if 1 ≤ *RQ_i_* < 10; and as high risk if *RQ_i_* ≥ 10 [57,58].

### 2.8. Statistical Analysis

Concentration data for air, soil, and water were processed using Microsoft Excel to determine descriptive statistics such as frequencies, means, and ranges. We used CalTOX for multimedia modeling. Principal Component Analysis (PCA) and Pearson’s correlation were used to explore possible relationships between the concentrations of pesticides in soil and water samples and the pesticide properties.

## 3. Results and Discussion

### 3.1. Pesticides in Soil and Water Samples

Data from the analyses of the soil and water samples are presented in Table 1. All of the analytes were detected in soil samples in the following ranges: 0.002–2.58 µg/g for ametryn, 0.003–0.19 µg/g for atrazine, 0.002–0.02 µg/g for pendimethalin, and 0.001–0.01 µg/g for 2,4-D (Table 1). Pesticide concentrations generally accumulate in greater concentrations in the topsoil compared to those found at lower soil levels [59], which would explain the high concentrations in our results. In regard to the pesticide composition of the soil samples, ametryn was the most abundant pesticide. In the irrigation water samples, the smallest amount was recorded for pendimethalin (in only 8% of the samples). Ametryn and atrazine were each detected in 33% of the samples, while 2,4-D was detected in 25% of the samples. Atrazine was found to have the highest concentrations in the water samples (0.104 µg/L). The mean concentrations of pesticide residues in the soil and irrigation water samples were weakly correlated with pesticide properties such as solubility in water and vapor pressure but strongly correlated with the dissociation constant (pKa) (see Figure S1 and Table S7).

Ametryn exhibited the highest mean concentrations in both the agricultural soil and irrigation water samples (0.89 µg/g and 0.02 µg/L, respectively). These results are consistent with our previous findings, in which ametryn consistently had the highest mean concentrations in all the analyzed samples (air, soil, and water) from the studied community [34,35]. Ametryn is moderately persistent, with a half-life (DT_50_) of 37 days [60] in soil and an aqueous hydrolysis half-life of 52.3 days [61]. Ametryn has low bioaccumulation due to its low octanol–water partition coefficient (Log P = 2.63). Studies have shown that the upper (surface) parts of streams and wells generally contain higher pesticide concentrations than the deeper waters [62,63]. However, our study did not collect groundwater samples for comparison with the pesticide residue levels in the surface water samples. Of all four pesticides investigated, pendimethalin had the lowest detectable traces in the water samples, which is not surprising in view of its low water solubility (0.33 mg/L), low leaching potential to move from soil into water bodies (GUS index = −0.28), and non-mobile quality (K_oc_ > 17,000). However, pendimethalin also had the highest octanol–water partition coefficient (Log P = 5.4) and the highest DT_50_ value (100.6 days), indicating that this compound has high persistence and bioaccumulation properties.

In the agricultural soil samples, ametryn had the highest concentration, and 2,4-D had the lowest. Our target compounds had no or low mobility, except for 2,4-D, which was moderately mobile (K_oc_ = 55.1). They also showed a moderate leaching potential (according to GUS index values), which could suggest a risk of groundwater pollution. In our study, the mean concentrations of pesticides in the soil samples tend to be higher than those from other studies from developing countries [62,64]. This is a potential health risk, as these pesticides may contaminate groundwater. The mean concentrations of our target compounds in the water samples were below the WHO maximum concentration limits for drinking water (Table 1). Pesticides may contaminate irrigation water through run-off, with water from the farm fields contaminating nearby irrigation water channels. The high potential of atrazine and ametryn for water contamination precipitated their removal from the European Union’s (EU’s) list of approved products (EC Regulation 1107/2009) in 2004 [60,65]. However, atrazine is still used in Canada, China, the USA, and most African countries [66,67]. Our results do not exceed the drinking water standards set by the EU (0.1 µg/L) and Canada (5 µg/L).

In comparison with reports from similar studies conducted in other developing countries, our mean concentrations in soil samples are higher than those in the data reported for Nepal and Ghana [62,64], although the pesticides studied do not match those used in this study. In contrast, the pesticide levels in our soil samples are lower than those in studies conducted in Iraq and Pakistan [68,69]. For water samples, our mean concentrations were generally at the lower end in comparison with studies conducted in Rwanda, Nigeria, Ghana, India, Thailand, and Ethiopia [62,63,70,71,72,73], as illustrated in Table 2.

### 3.2. Health Risk Assessments

**Figure 3:**
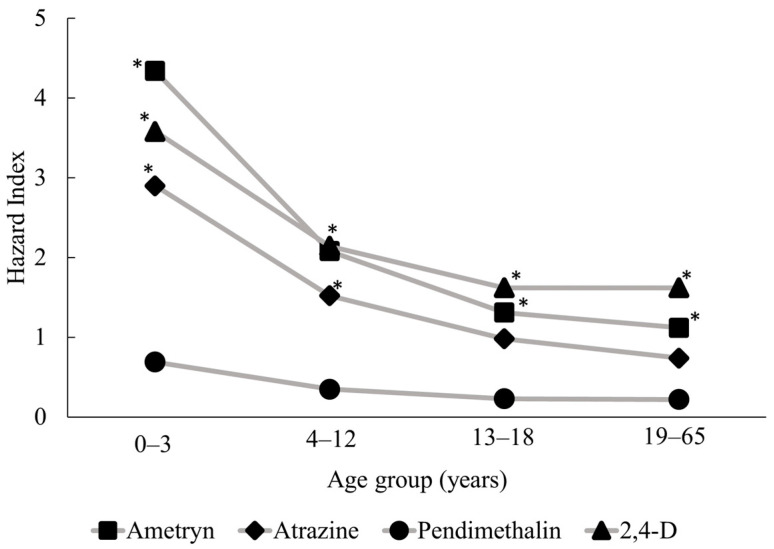
Hazard Index values at the 95th percentile show the multi-pathway risk of exposure to the four target pesticides for each age group. * Hazard Index > 1.

Following the probabilistic risk assessment, we examined HI distributions representing non-carcinogenic risks associated with multi-pathway and multi-route exposure for each of the four pesticides in all age groups (see Table 3). Residents in the study community are most likely to be exposed to ametryn and least likely to be exposed to pendimethalin, as ametryn consistently had the highest mean concentrations in all environmental media. Children aged 0–3 years had the highest risk of exposure for all target compounds, with 95th percentile total risk values (HI_95_) for ametryn, atrazine, pendimethalin, and 2,4-D of 4.34, 2.90, 0.69, and 3.58, respectively (Figure 3). HI_95_ values exceeded 1 for ametryn, atrazine, and 2,4-D, which suggests that the contamination of various environmental media with these pesticides could pose significant risks to the residents of the study area, particularly through the ingestion of water and locally produced fruit and vegetables. When pesticides are applied in fields, a fraction of the applied dose is emitted into the air through volatilization and spray drift [12,74]. Most of pesticides applied are deposited onto the soil surface and contaminate the irrigation water bodies through agricultural run-off. When climate change causes a significant drop in precipitation, irrigation water is the main driving factor of run-off contamination [74].

For all of our target compounds, exposure through ingestion was the most dominant pathway, as it had the highest contribution (Table 3). Dietary intake, in the form of local fresh produce, was the most important contributor to the total ingestion dose. Indirectly exposed produce (i.e., meat, eggs, etc.) had a relatively lower contribution to the ingestion dose. Our study area is an intensive farming area that not only grows sugarcane but also fruit and vegetables. Thus, it is reasonable to expect a high share of local produce in the dietary intake of the residents in the study community. Due to the high pesticide levels in the soil compartment reported in this study, the highest exposure levels in our analysis were derived from the soil. Plants are mainly contaminated through the soil [75], and ingesting locally grown produce is a significant contributor to the overall risk of pesticide exposure. In our analysis, the average daily doses of young children (0–3 years) were more than twice those of adults (19–65 years). These results are similar to those reported in previous studies conducted in the USA [6,75], in which the average daily doses and risks of exposure amongst children were higher than those of adults (1.6× and 2×, respectively). We found that pendimethalin had the lowest average ratio of concentration of the reference dose, so this pesticide poses the lowest risk of exposure, as the total risk values for all age groups were acceptable (HI_95_ < 1). Even though the amount of pendimethalin used was not the lowest, compared with the other target pesticides, pendimethalin has the lowest solubility in water and the lowest potential to leach and is non-mobile. Thus, a very small amount of the pesticide is absorbed into the ground and, subsequently, into the irrigation water through run-off.

**Figure 4:**
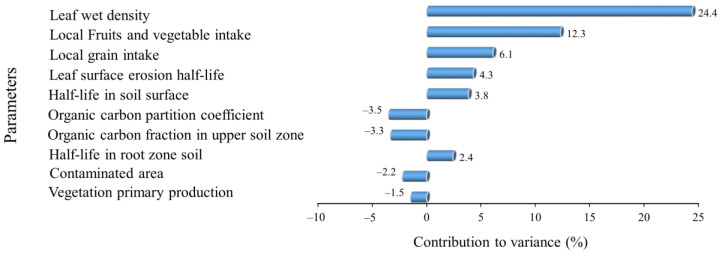
The top 10 parameters identified in the sensitivity analysis.

None of the studies conducted in developing countries cited in this paper conducted health risk assessments for local residents. However, the high concentrations of pesticide residues in the soil and water samples could suggest a high health risk, as our study and others [6,75] show that the ingestion pathway is the most dominant contributor to the total risk. Our risk assessment analysis only considered exposure to our four target compounds contributed by the analyzed environmental media. To determine the relative changes in total risk due to changes in the model inputs, we performed a sensitivity analysis. Due to our target compounds being present mainly in soil and water and their transportation through environmental media, our sensitivity analysis indicates that health risks arise from human exposure parameters and the characteristics of the target compounds; the environmental parameters in the model are comparatively less sensitive (Figure 4). This implies that the amounts of pesticides used has a significant impact on the total risk, as pesticides’ properties such as their half-life in soil are sensitive. The dietary intake of local produce was sensitive, as it had the highest contribution to the total risk.

### 3.3. Ecological Risk Assessment

Eswatini is a landlocked country, i.e., it has no access to the ocean, so freshwater fishing is the only option for communities. To evaluate the impact of the four studied pesticides on aquatic organisms (fish and aquatic waterbirds), we calculated the RQ using the AF method, incorporating the HC_50_ values obtained from the toxicity database. As shown in Table 4, these pesticides were associated with negligible acute and chronic risks for both fish and aquatic invertebrates, indicating minimal risk. A study conducted in Rwanda has found that malathion poses a risk to arthropods (RQ > 1), and the persistence of this pesticide may lead to a loss of biodiversity in the fish and aquatic invertebrate community [63].

Climate change is responsible for extreme weather events, such as droughts and storms, which may affect the environmental distribution of pesticides [76]. Environmental factors that influence the fates of pesticides include precipitation, temperature, wind, and carbon dioxide levels. Due to the effects of climate change, these environmental factors are changing and will continue to influence the deposition and absorption of pesticides [74]. More studies are needed to explore the impact of climate change on environmental pesticide distribution and toxicity.

### 3.4. Limitations of This Study

This study provides evidence that pesticide contaminants in soil and recycled irrigation water may pose significant health risks for residents in agricultural areas, mainly through food consumption. We did not analyze pesticide residues in locally grown food produce. The long-term monitoring of pesticide residues in locally produced crops when assessing dietary exposure to pesticides would reduce uncertainty and improve the quality of the study data. This study used the US California EPA’s CalTOX model to assess pesticide exposure. The study area yielded insufficient local environmental data. Thus, as mentioned earlier in this paper, we used data from Arizona in the USA for some environmental parameters, which is potentially a cause for uncertainty in our analysis. If the environmental parameters used in the CalTOX model were to be collected in Eswatini, the uncertainty would be reduced. The CalTOX model requires further validation in relation to Eswatini. Moreover, we only focused on four commonly used pesticides, while the community is exposed to other pesticides, so we may have underestimated the risks presented in this study. Lastly, our irrigation water samples had the smallest detection frequencies in comparison with other environmental media, with the highest frequency at 33% and the lowest at 8%.

## 4. Conclusions

In this study, we selected an agricultural community in Eswatini as an example of a country in Southern Africa facing the impacts of climate change on the health and ecological risks arising from agricultural pesticide exposure through recycled irrigation water and farmland soil. Our analysis combined on-site sampling and a multi-pathway and multi-route exposure modeling approach. Our probabilistic health risk assessment of commonly used ametryn, atrazine, and 2,4-D has revealed that these pesticides pose significant health risks for community residents in agricultural areas in Eswatini. Although we cannot directly compare our data with those presented in analyses of pesticide use for a number of other developing countries in Africa, Asia, and the Middle East, all of which have used pesticides different from those in our study, we suspect that the pesticide residues in the soil and water systems of those countries may pose health risks for their citizens. Our findings can be used for behavioral and regulatory interventions to better protect residents in agricultural areas in Eswatini and in many other countries throughout Africa from health risks linked to pesticide exposure. The focus of research should be on the ingestion pathway of exposure through the soil and the consumption of local produce, as these were the most dominant sources of exposure. These developing countries have a mandate and duty to deliver on their commitments regarding the UN Sustainable Development Goals and to provide clean and safe drinking water, particularly to rural agricultural communities. Importantly, since the pesticide-based contamination of agricultural soil is linked to food safety concerns, more studies are needed to analyze pesticide residues in food products from agricultural communities. This study is the first step in the assessment of the health risks of pesticide exposure for agricultural communities in Eswatini. Further, epidemiological research should be focused on scientifically evaluating the association between exposure to pesticides and specific adverse health outcomes in rural agricultural communities in Eswatini.

## Figures and Tables

**Table 1:** Mean concentrations of pesticide residues in soil and water samples.

	Soil Samples (µg/g) *n* = 10 ^a^		Water Samples (µg/L) *n* = 12 ^b^		
Target Compounds	Range	Mean ± SD	Range	Mean ± SD	WHO MCLs ^c^
Ametryn	0.002–2.58	0.89 ± 0.28	0.0004–0.1	0.02 ± 0.03	–
Atrazine	0.003–0.19	0.03 ± 0.02	0.0003–0.104	0.02 ± 0.04	2
Pendimethalin	0.002–0.02	0.01 ± 0.002	0.001–0.004	0.001 ± 0.001	20
2,4-D	0.001–0.01	0.004 ± 0.001	0.001–0.005	0.002 ± 0.002	3

^a^ Detection rates of analytes (60–100%); ^b^ detection rates of analytes (8–33%); ^c^ World Health Organization’s maximum concentration limits (MCL) for drinking water (µg/L) [60].

**Table 2:** A comparison of pesticide levels in water and soil samples in this study with those in published data for other developing countries.

Country	Target Compounds	Mean Concentrations		References
		Soil Samples (µg/g)	Water Samples (µg/L)	
Eswatini	4 pesticides	0.004–0.89	0.001–0.02	This study
Pakistan	11 pesticides	0.09–1.39	–	[68]
Nepal	15 pesticides and degradates	1.34 × 10^−3^–0.041	–	[64]
Rwanda	33 pesticides	–	ND–4.82	[63]
Nigeria	16 pesticides and degradates	–	ND–1.65	[73]
India	7 pesticides	–	ND–0.44	[71]
Ghana	3 pesticides	0.01–0.04	ND–0.05	[62]
Thailand	3 pesticides	–	0.09–0.42	[70]
Ethiopia	4 pesticides	–	0.11–138	[72]
Iraq	2 pesticides	0.21–1.62	–	[69]

ND = not detectable.

**Table 3:** Average daily doses (µg/kg/day) at the 95th percentile for different age groups. Exposure pathways were estimated using the CalTOX model.

Pesticides	Inhalation	Ingestion			Dermal	Total
		Exposed Produce	Unexposed Produce	Water		
0–3 years						
Ametryn	0.415	5.180	1.070	0.010	0.001	6.680
Atrazine	0.159	5.750	0.020	1.200	0.080	7.210
Pendimethalin	0.294	2.720	0.220	1.8 × 10^−5^	4.4 × 10^−5^	3.750
2,4-D	0.004	7.700	0.063	0.014	0.001	7.790
4–12 years						
Ametryn	0.256	2.570	0.535	0.003	0.005	3.370
Atrazine	0.100	2.850	0.010	0.479	0.242	3.680
Pendimethalin	0.181	1.620	0.132	7.0 × 10^−6^	1.0 × 10^−4^	2.450
2,4-D	0.002	3.820	0.031	0.005	0.004	3.870
13–18 years						
Ametryn	0.171	1.730	0.357	0.003	0.009	2.270
Atrazine	0.065	1.920	0.006	0.479	0.483	2.950
Pendimethalin	0.121	1.090	0.088	7.0 × 10^−6^	2.6 × 10^−4^	1.810
2,4-D	0.002	2.570	0.021	0.005	0.007	2.610
19–65 years						
Ametryn	0.155	1.500	0.357	0.003	0.011	2.040
Atrazine	0.060	1.670	0.006	0.046	0.564	2.760
Pendimethalin	0.112	1.230	0.016	5.7 × 10^−6^	0.002	2.030
2,4-D	0.001	2.240	0.021	0.005	0.008	2.280

**Table 4:** Risk quotient values (95th percentiles) for the target compounds.

	Fish		Aquatic Invertebrates	
Pesticides	Acute (96 h)	Chronic (21 Days)	Acute (48 h)	Chronic (21 Days)
Ametryn	4.0 × 10^−5^	2.9 × 10^−4^	7.1 × 10^−6^	6.3 ×10^−4^
Atrazine	4.4 × 10^−5^	1.0 × 10^−4^	2.4 × 10^−6^	8.0 × 10^−4^
Pendimethalin	5.1 × 10^−5^	2.0 × 10^−3^	6.8 × 10^−5^	6.9 × 10^−4^
2,4-D	2.0 × 10^−7^	7.4 × 10^−7^	1.5 × 10^−7^	4.3 × 10^−7^

## Data Availability

The data that support the findings of this study are available on request from the corresponding author.

## Supplementary Materials

The following supporting information can be downloaded at https://www.mdpi.com/article/10.3390/toxics11090770/s1, Figure S1. Principal component analysis of pesticide concentrations in soil and water samples and the pesticides’ properties. Mean and maximum concentrations of pesticides in soil and water samples were strongly correlated with the dissociation constant at 25 °C (Dis.) but weakly related to the other properties. DT_50_, soil half-life time; K_oc_, organic carbon–water partition coefficient (mL/g); Aq. hydrolysis, aqueous hydrolysis of pesticides; VP, vapor pressure at 25 °C (mPa); Log P, octanol–water partition coefficient at pH7 and 20 °C; Solubility, solubility in water at 20 °C (mg/L); Groundwater Ubiquity Score (GUS) index, leaching potential; Table S1. Properties of the target pesticides [77]; Table S2. Mean recoveries of soil and water samples; Table S3. Pesticides with their cancer classifications, acceptable daily intake (ADI), reference dose (RfD), and human health effects; Table S4. Pesticide concentrations in different environmental media (air, soil, and water) from samples collected in Eswatini; Table S5. Parameters of landscape properties used in our analysis; Table S6. Parameters for each age group and their respective values for non-carcinogenic risk assessment; Table S7. Correlations between the parameters and pesticide mean and maximum concentrations.
