# Supplementary material for: Impacts of Agricultural Pesticide Contamination: An Integrated Risk Assessment of Rural Communities of Eswatini
Source: Toxics. 2023 Sep 10;11(9):770. doi: 10.3390/toxics11090770 (PMC10534646; doi:10.3390/toxics11090770)
Supplement: Supplementary file 1 [file toxics-11-00770-s001.zip › toxics-2586302-supplementary.pdf]

SUPPLEMENTARY INFORMATION

Table S1. Properties of the target pesticides.

Target pesticides	Chemical group	Molecular formula	Molecular weight (g/mol)	Vapor pressure, 20°C (mPa)	Henry's law constant (Pa m³ mol⁻¹)	[a]K_{oc} (mg/L)	[b]Log P, pH 7, 20°C	Solubility in Water, 20°C (mg/L)	Dissociation constant at 25°C	[c]DT_{50} (field days)	Aqueous hydrolysis (days)	GUS index
Ametryn	Triazine	$C_9H_{17}N_5S$	227.33	0.37	4.1×10^{-4}	316	2.6	200	10.07	37	-	0.46
Atrazine	Triazine	$C_8H_{14}ClN_5$	215.68	0.04	1.5×10^{-4}	100	2.7	70	1.7	29	86	2.57
Pendimethalin	Dinitroaniline	$C_{13}H_{19}N_3O_4$	281.31	3.34	1.27	17491	5.4	0.33	2.8	100	21	-0.28
2,4-D	Alkylchlorophenoxy	$C_8H_6Cl_2O_3$	221.03	0.01	4.0×10^{-6}	55.1	2.8	900	3.4	28.8	38	3.82

[a] Soil organic carbon-water partitioning coefficient; [b] Octanol-water partition coefficient; [c] Half-life time.

References: Pesticide Properties DataBase [60]; Hazardous Substance Databank [77].

Table S2. Mean recoveries of soil and water samples.

	Soil samples		Water samples	
**Target compounds**	**Mean Recovery (%)**	**%RSD**	**Mean Recovery (%)**	**%RSD**
Ametryn	105.5	5.8	72.8	21.8
Atrazine	140.4	3.6	112	22.7
Pendimethalin	118.4	9.3	61.9	6.6
2,4-D	110.4	0.7	130.1	35.5

Table S3. Pesticides with their cancer classifications, acceptable daily intake (ADI), reference dose (RfD), and human health effects.

**Target compounds**	**EPA carcinogenic potential classification**[a]	**RfD (mg/kg/day)**[b]	**Human health effects**[b]
Ametryn	Suggestive evidence of carcinogenic potential	0.009	Eye irritant
Atrazine	Not likely to be carcinogenic to humans	0.035	Skin irritant<br>Endocrine disruptor
Pendimethalin	Group C—Possible human carcinogen	0.04	Bioaccumulates<br>Thyroid and liver toxicant
2,4-D	Group D—Not classifiable as to human carcinogenicity	0.01	Respiratory tract irritant<br>Reproductive/developmental effects

ADI: Average daily intake; RfD: Reference dose.

[a] USEPA Chemicals Evaluated for Carcinogenic Potential (Annual Cancer Report 2022) [42].

[b] Pesticide Properties DataBase (PPDB)[60].

Table S4. Pesticide concentrations in different environmental media (air, soil, and water) from samples collected in Eswatini.

	**Mean concentrations**				
	**Ametryn**	**Atrazine**	**Pendimethalin**	**2,4-D**	**References**
**Personal air samples (μg/m³)**	36.91	21.57	31.05	0.89	[34]
**Household indoor air (μg/m³)**	0.47	0.18	0.30	0.004	[35]
**Soil (μg/g)**	0.89	0.03	0.01	0.004	This study
**Water (μg/L)**	0.02	0.02	0.001	0.002	This study

Table S5. Parameters of landscape properties used in our analysis.

Landscape properties	Mean value	Coefficient of variation (CV)	Reference
Contaminated area (m^2)	1.40E+05	0.10[a]	According to applicators (personal communication, October 2018)
Annual average precipitation (m/day)	1.10E-03	0.33	Average weather in Vuvulane[b]
Ambient environmental temperature (K)	2.94E+02	0.10	Average weather in Vuvulane[b]
Annual average wind speed (m/day)	3.54E+05	0.13	Average weather in Vuvulane[b]

m: meters

[a]The CV was set at 0.1 if the standard deviation was unknown.

[b] http://weatherspark.com

Table S6. Parameters for each age group and their respective values for non-carcinogenic risk assessment.

Parameters / Age groups	0–3 years	4–12 years	13–18 years	19–65 years	References
Body weight (kg)	1.38E+01	3.18E+01	5.68E+01	6.81E+01	[a]USEPA [48]; [b]DRFSR [49]
Surface area (m^2/kg bw)	1.00E-02	3.00E-02	6.00E-02	7.00E-02	USEPA [48]
Active breathing rate (m^3/kg bw/h)	8.00E-02	5.00E-02	3.30E-02	3.00E-02	USEPA [48]
Resting breathing rate (m^3/kg bw/h)	2.70E-02	1.60E-02	1.10E-02	1.00E-02	USEPA [48]
Fluid intake (L/kg bw/day)	5.00E-02	2.20E-02	2.20E-02	1.90E-02	CalTOX
Fruit and vegetable intake (kg/kg bw/day)	1.20E-02	6.00E-03	4.00E-03	4.00E-03	CalTOX
Grain intake (kg/kg bw/day)	1.20E-02	5.90E-03	4.000E-03	3.00E-03	CalTOX
Milk intake (kg/kg bw/day)	6.50E-03	6.50E-03	6.50E-03	6.50E-03	CalTOX
Meat intake (kg/kg bw/day)	3.00E-03	3.00E-03	3.00E-03	3.00E-03	CalTOX
Egg intake (kg/kg bw/day)	4.60E-04	4.60E-04	4.60E-04	4.60E-04	CalTOX
Fish intake (kg/kg bw/day)	2.90E-04	2.90E-04	2.90E-04	2.90E-04	CalTOX
Soil ingestion (kg bw/day)	3.50E-07	3.50E-07	3.50E-07	3.50E-07	CalTOX
Breast milk ingestion by infants (kg/kg bw/day)	1.10E-01	1.10E-01	1.10E-01	1.10E-01	CalTOX
Exposure duration (years)	6.00E+01	6.00E+01	6.00E+01	6.00E+01	[b]DRFSR [49]
Average time (days)	2.19E+04	2.19E+04	2.19E+04	2.19E+04	[b]DRFSR [49]

[a] USEPA. Exposure Factors Handbook: 2011 Edition (2011)[48].
[b] Eswatini Government. Noncommunicable Disease Risk Factor Surveillance Report (2014)[49].

Table S7. Correlations between the parameters and pesticide mean and maximum concentrations.

Parameters	Mean (W)	Max (W)	Mean (S)	Max (S)	Solubility	K_{oc}	VP	Aq. hydro.	DT_{50}	Dis.	Log P	GUS index
**Mean (W)**	1											
**Max (W)**	**0.999**	1										
**Mean (S)**	**0.597**	**0.574**	1									
**Max (S)**	**0.627**	**0.605**	**0.999**	1								
**Solubility**	-0.542	-0.568	-0.341	-0.359	1							
**K_{oc}**	-0.601	-0.577	-0.331	-0.347	-0.345	1						
**VP**	-0.560	-0.537	-0.247	-0.264	-0.389	**0.996**	1					
**Aq. hydro.**	0.513	**0.545**	-0.333	-0.298	-0.530	-0.081	-0.111	1				
**DT_{50}**	-0.559	-0.536	-0.237	-0.255	-0.389	**0.995**	**1.000**	-0.120	1			
**Dis.**	**0.428**	**0.400**	**0.978**	**0.970**	-0.186	-0.287	-0.205	-0.523	-0.194	1		
**Log P**	0.047	0.078	0.044	0.048	-0.865	**0.70**	**0.796**	0.327	**0.796**	-0.038	1	
**GUS index**	-0.044	-0.058	-0.415	-0.407	**0.768**	-0.688	-0.746	0.083	-0.750	-0.388	-0.885	1

Positive correlations are in bold.

Mean (W): mean concentration of surface water samples for each analyte; Max (W): maximum concentration detected in surface water samples for each analyte; Mean (S): mean concentration of water samples for each analyte; Max (S): maximum concentration detected in soil samples for each analyte; Solubility: solubility in water, 20°C; K_{oc}: soil organic carbon-water partitioning coefficient; VP: vapor pressure; Aq. hyd.: aqueous

hydrolysis; DT_{50}: half-life time (field days); Dis.: Dissociation constant at 25°C; Log P: Octanol-water Partition Coefficient; GUS index: leachability of compound.

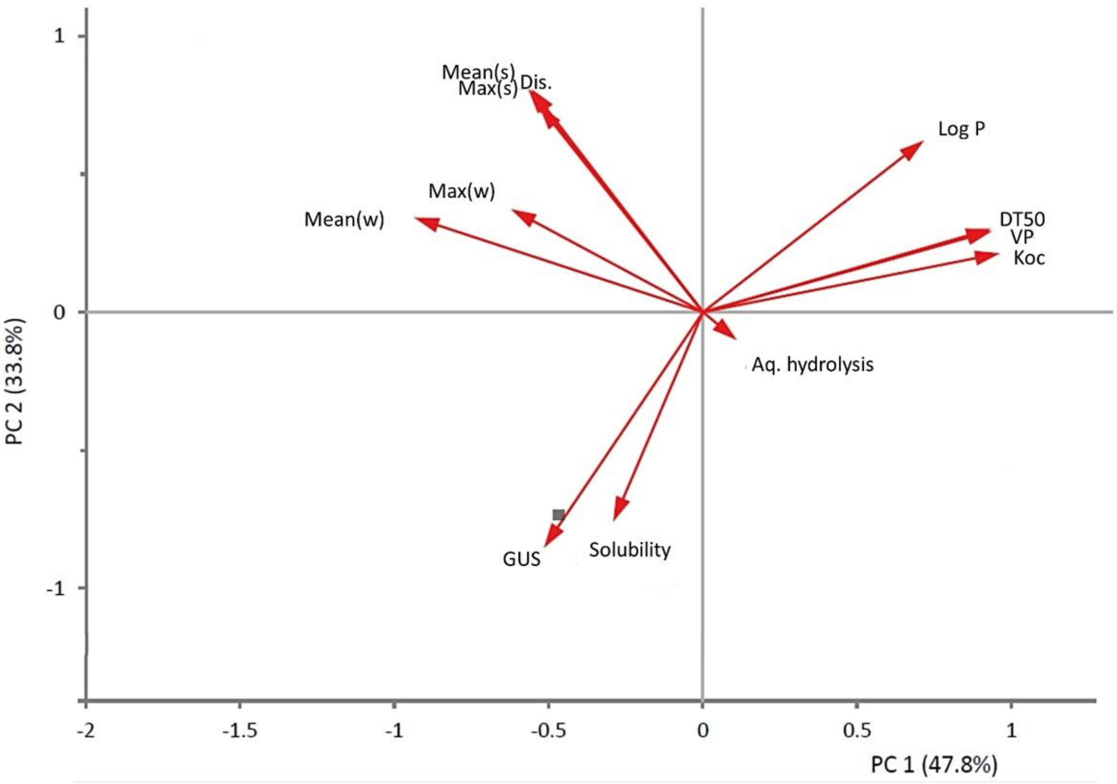

Figure S1. Principal component analysis of pesticide concentrations in soil and water samples and the pesticides' properties. Mean and maximum concentrations of pesticides in soil and water samples were strongly correlated with the dissociation constant at 25°C (Dis.) but weakly related to the other properties. DT_{50}, soil half-life time; K_{oc}, organic carbon-water partition coefficient (mL/g); Aq. hydrolysis, aqueous hydrolysis of pesticides; VP, vapor pressure at 25°C (mPa); Log P, octanol–water partition coefficient at pH7 and 20°C; Solubility, solubility in water at 20°C (mg/L); Groundwater Ubiquity Score (GUS) index, leaching potential.
